# New insights on sea turtle behaviour during the ‘lost years’

**DOI:** 10.1098/rspb.2024.2367

**Published:** 2025-02-05

**Authors:** Katrina F. Phillips, Nathan F. Putman, Katherine L. Mansfield

**Affiliations:** ^1^ Department of Biology, University of Central Florida, Orlando, Florida, USA; ^2^ LGL Ecological Research Associates, Bryan, Texas, USA

**Keywords:** juvenile dispersal, satellite telemetry, ontogeny

## Abstract

Several marine turtle species spend their first years of life in oceanic habitats. This early life stage is referred to as the ‘lost years’ due to the difficulty of accessing individuals for study offshore. We satellite tracked 114 wild-caught juvenile turtles (straight carapace lengths 12.3–29.9 cm) from the Gulf of Mexico between 2011 and 2022 to investigate ‘lost years’ movements with respect to traditional definitions assigned to the life stage. Satellite-tracked turtles included 79 green turtles (*Chelonia mydas*), 26 Kemp’s ridleys (*Lepidochelys kempii*), 5 loggerheads (*Caretta caretta*) and 4 hawksbills (*Eretmochelys imbricata*). Many tracked turtles transited between oceanic (>200 m depth) and neritic waters (<200 m depth), challenging the assumption that this life stage is exclusively found in oceanic habitats. Turtle movements differed from oceanographic surface drifters, providing further evidence that sea turtles of this life stage do not exclusively drift with currents. We recommend redefining the ‘oceanic stage’ as a ‘dispersal stage’ to better reflect their behaviour and habitat plasticity. Our findings establish the West Florida Shelf as a high-use area, particularly among green turtles and Kemp’s ridleys. The northeastern Gulf of Mexico is an important region for these species of conservation concern.

## 1. Introduction

Juvenile dispersal occurs across taxa, often facilitated by environmental variables such as wind and water [1–3]. The resulting dispersal trajectories deliver individuals to a range of potential habitat types and available resources. Juvenile or larval dispersal might be direct, with settlement and recruitment to a single location (e.g. plants and corals), or more complex, with one or many ontogenetic habitat shifts over time (e.g. amphibians, marine invertebrates, salmon, eels, sea turtles). These shifts may have evolved in response to a number of factors, such as predator avoidance and optimizing the resources or conditions for growth [4]. Regardless of the mechanism, juvenile dispersal leads to the distribution of individuals across habitats and life stages.

In four of the seven sea turtle species—green turtles (*Chelonia mydas*), Kemp’s ridleys (*Lepidochelys kempii*), loggerheads (*Caretta caretta*) and hawksbills (*Eretmochelys imbricata*)—juvenile stages are defined by individual size and the habitat type where they are found: terrestrial, oceanic or neritic [5–7]. The other three species are primarily neritic (*Natator depressus*) or oceanic (*Dermochelys coriacea, Lepidochelys olivacea*) throughout their life cycle [5, 8]. Early hypotheses about sea turtle life history and ontogeny across most species include: (i) the early juvenile stage includes several years in exclusively ‘oceanic’ environments, defined as water off the continental shelf >200 m depth [5–7]; (ii) oceanic-stage juveniles drift passively with currents [9]; and (iii) life stage transitions are discrete, with unidirectional recruitment to the next habitat type. Direct measurements to test these hypotheses were previously limited to labour-intensive visual observations or radio telemetry with limited tracking time and distance from shore (10–15; reviewed by Mansfield & Putman [16]). Advances in telemetry technology have reduced satellite tag size using solar-charging batteries, which facilitated the first long-term satellite tracks of small, oceanic-stage sea turtles (e.g. [17–21]). Mansfield *et al*. [18,20] satellite-tracked laboratory-reared loggerhead and green turtles over 4000 km from their release location and demonstrated that not all juveniles behave as historically hypothesized. While turtles released from southeast Florida travelled offshore of the continental shelf and probably remained at the sea surface, many turtles exited currents associated with the North Atlantic Subtropical Gyre, travelling into the centre of the Gyre and into the Sargasso Sea, exhibiting directional movement [18,20].

Oceanic juvenile sea turtle movements are a combination of transport through ocean currents and probably guided in part by swimming behaviour [21–23]. Ocean circulation models reasonably depict broadscale aspects of juvenile sea turtle distributions [24–26], but without representing swimming behaviour, they can fail to account for finer scale observations and ecological processes [16,21,22,27,28]. Studies that compared turtle movements to oceanographic drifters suggest that swimming by young sea turtles directs their oceanic dispersal [19,21]. In addition, research in the Gulf of Mexico and South Atlantic suggests that young oceanic-stage turtles actively swim and there may be distinct differences among species in orientation, dispersal and swimming behaviour [19,21]. Understanding whether and when juvenile turtles are active swimmers or passive drifters is critical for determining dispersal trajectories, exposure and transition times in areas impacted by anthropogenic activities (e.g. oil spills) and connectivity between populations and habitats [29]. Without basic biological and behavioural data, it is difficult to improve assessments of anthropogenic impacts on protected species, necessitating conservative or precautionary approaches to management using ‘best available data’.

Juvenile sea turtles found in the eastern Gulf of Mexico are predicted to be close in size and age to turtles transitioning from oceanic to neritic life stages [30]. Developmental foraging habitats for larger, coastal-stage juveniles are known to occur along the West Florida Shelf and the northeastern Gulf of Mexico [31–38]. The transition from oceanic to neritic habitats is tied to a shift in target prey items, from omnivory at the sea surface [39] to more specialized benthic foraging that differs for each species [40]. Green turtles generally grow faster in width than loggerheads, and recruit nearshore at smaller straight carapace lengths (SCLs) [41]; size at recruitment to nearshore habitats occurs around 20−30 cm SCL for green turtles, Kemp’s ridleys and hawksbills, while loggerheads are more often over 40 cm SCL (electronic supplementary material, table S1). However, neritic recruitment may not be a discrete shift, as larger recruiting loggerheads have shown plasticity in habitat selection with repeated shifts between neritic and oceanic foraging areas [42–45]. Similar tracking data are limited for juvenile green turtles, hawksbills and Kemp’s ridleys, but diet data suggest plasticity in nearshore recruitment, with a transitional stage between carnivory and herbivory in recently recruited green turtles [40,46,47]. Additionally, juvenile green turtles may first recruit to hard-bottom algae-dominated habitats and later shift to seagrass habitats [48–52].

The environmental impact assessment response to the 2010 *Deepwater Horizon* oil spill documented thousands of small turtles in the northern Gulf of Mexico, with estimates of oceanic-stage turtles killed ranging from 55 000 to 159 000 [53,54]. The high uncertainty around this estimate is due in part to a lack of baseline information on the abundance and distribution of small individuals in oceanic habitats, which were particularly impacted by the spill because they tend to stay near the surface and frontal boundaries [30,53,55,56]. In comparison, mortality estimates for larger neritic juveniles and adults ranged from 4900 to 7600 [54]. An additional data gap was identifying the regions where juveniles in the Gulf of Mexico originate. Genetic analyses and ocean circulation model predictions suggest that the majority of oceanic-stage juvenile sea turtles in the northeastern Gulf of Mexico may originate in Mexico [29,30,32,57–59], but the genetic resolution and spatial coverage of the data leaves high levels of uncertainty.

The goals of this study were to: (ii) characterize early juvenile sea turtle movements and assess turtle movements relative to the traditional definitions of sea turtle ontogenetic shifts; (ii) compare turtle movements to passive oceanographic drifter movements for evidence of swimming behaviour; and (iii) examine potential neritic recruitment to better describe turtle sizes and locations where ontogenetic transitions between oceanic and neritic habitats occur. This study fills multiple data gaps regarding the movements and behaviours of the ‘lost years’ life stage; the distribution of small juvenile sea turtles in the Gulf of Mexico; the degree to which currents influence turtle movements; and the turtle sizes at which ontogenetic habitat transitions occur.

## 2. Methods

### (a) Turtle capture and tag attachment

We captured 131 small juvenile sea turtles in the Gulf of Mexico in 2011−2022: green turtles (*n* = 94), Kemp’s ridleys (*n* = 28), loggerheads (*n* = 5) and hawksbills (*n* = 4). Search areas were 35−200 km offshore from boat launch sites in Venice, Louisiana (USA) and Sarasota, Cortez and Destin, Florida (USA). We travelled offshore from each port until we located floating clumps and lines of seaweed in the genus *Sargassum*. We searched *Sargassum* habitats for small turtles, and once spotted, we navigated to the turtle and captured it using a modified long-handled dip net [60]. Once on board, we collected carapace measurements, skin and blood samples [29] and weights.

To track post-release movements, we affixed Microwave Telemetry 9.5 g solar-powered platform transmitter terminals (PTTs) to the turtles. Seventeen sampled turtles were not satellite-tracked due to tag availability, permit limitations or tags that failed to transmit after turtles were released. For all species, we cleaned and sanded the carapace with isopropyl alcohol swabs and 120-grit sandpaper prior to tag application [17,60]. For Kemp’s ridleys (*n* = 26), loggerheads (*n* = 5) and hawksbills (*n* = 4), we pre-treated carapaces with manicure acrylic, then attached two narrow strips of 3−5 mm neoprene to either side of the turtle’s vertebral ridge using veterinary or toupée/hair extension glue [17,18,60]. We used clear aquarium silicone to affix the tag to the neoprene and carapace and shaped the attachment into a hydrodynamic teardrop shape smoothing the silicone as it set [17,18,60]. For green turtles (*n* = 79), the attachment method differed, and we used a flexible marine adhesive 3M 4200 or 5200 [20,21,60]. Both attachment methods result in a flexible base, allowing for some growth before shedding naturally with scutes [17,18,20]. Dispersing juvenile sea turtles are predominantly surface-dwelling; tags and antennae are often exposed to air, thereby minimizing hydrodynamic drag associated with the tag attachment site, ensuring regular communication with overhead satellite-mounted Argos receivers for position estimates, and charging of the tag’s solar cells [17,18,20]. The PTTs had a 10 h on/48 h off-duty cycle to facilitate solar charging. In addition to PTTs, we inserted subdermal passive integrated transponders (PIT tags) in the right front flipper muscle. We released all turtles together at the end of each sampling day, near their capture site and into the *Sargassum* habitat.

### (b) Surface drifters

We deployed oceanographic surface drifters with each group of turtles released to compare movements of known passive objects against turtle movements [21]. We refer to each release of turtles and drifters as a deployment, with a total of 37 deployments between 2011 and 2022 (electronic supplementary material, tables S2 and S3). We typically released a pair of drifters at each deployment with the goal of comparing the divergence between drifters to the divergence between drifters and turtles. Drifters were unavailable for deployments 3, 6 and 16−18. For deployments 1−2, 4−5 and 7−15, we deployed paired ‘Kathleen’ (ballasted 5-gallon buckets that extended from the surface to 37 cm) and ‘Eddie’ (1 m drogue) drifters as described by Putman and Mansfield [21]; for deployments 19−23, we deployed paired ‘Kathleen’ drifters; and in 2017, we transitioned to biodegradable CARTHE (Consortium for Advanced Research on Transport of Hydrocarbon in the Environment) drifters with a drogue at 40 cm below the surface [61]. This included two deployments of paired ‘Kathleen’/CARTHE drifters (deployments 24−25) and the rest paired CARTHE drifters (deployments 26−37). All drifters relayed GPS positions, with ‘Kathleen’ and ‘Eddie’ drifters programmed to relay locations every 1 h in 2012 and every 30 min for the remaining years, and the CARTHE drifters transmitting data at 5 min intervals.

### (c) Data analysis

To analyse differences between turtle and drifter movements, we evaluated drifter and turtle data separately using the bsam package in R [62–64]. We used a Bayesian hierarchical first difference correlated random walk model (CRW) to interpolate positions at 12 h intervals, and the Gelman–Reubin shrink factor to check for convergence. The CRW analysis was followed by a hierarchical switching state-space model (SSM) that uses a combination of speed and sinuosity to assign movement along a scale of two states: *state 1* is fast and directed movements while *state 2* is slower and more meandering [62,63]. We removed data for two turtles from the SSM analysis: one green turtle with an 8 h tracking duration shorter than the 12 h model timestep, and one green turtle with a 7 day gap between locations that failed to converge. Similarly, we trimmed any drifter tracks with a gap between locations of 7 days or more to end before the start of the transmission gap. Given the gaps in telemetry data due to duty cycles, two of the interpolated tracks for turtles moving quickly with the Gulf Stream current failed to accurately estimate positions and appeared to cross over land in southern Florida; we removed interpolated locations that occurred on land for this loggerhead and green turtle as well as any related points that diverged significantly from other interpolated turtle positions.

Putman & Mansfield [21] previously analysed distances between drifters and turtles from 2011 to 2014 (deployments 1−15); here, we increase the original sample sizes, including those deployments as well as deployments in 2015, 2016, 2017, 2021 and 2022 (deployments 16−37). For drifter and turtle divergence analysis, we used interpolated turtle tracks from the SSM for consistent timesteps and the full drifter dataset. We calculated the time since deployment for each turtle and drifter position and selected the datapoint closest to 1, 3, 5, 7, 9, 11 and 13 days post-deployment. We then paired each drifter and turtle within a deployment to calculate the distance between them at each timestep. As an additional comparison between turtle and drifter movements, we paired raw drifter locations and SSM turtle data with 0.25° resolution hourly sea surface temperature data (ECMWF-ERA5) using bilinear interpolation in the ENV-DATA tool on Movebank [65]. To compare the temperatures at turtle and drifter locations, we fit a hierarchical generalized additive model [66,67] to the relationship between day of the year and temperature. The model included a global thin plate spline as well as separate splines for turtles and drifters to test for differences in temperatures across the year, with year included as a random effect [66].

We defined the 200 m depth contour as the edge of the continental shelf to assess turtle and drifter locations over the continental shelf (neritic) and off the shelf (oceanic) [6,7]. To better estimate potential movements towards nearshore recruitment that would include a transition from the sea surface to interaction with benthic habitats, we analysed a subset of the dataset, including only locations within 20 km of the coastline, a more spatially restricted area for the continental shelf that generally extends 20−200 km from shore in this region. This subset of the data within 20 km of shore was used as a proxy for potential recruitment to neritic habitats.

## 3. Results

### (a) Turtle data

**Figure 1.**
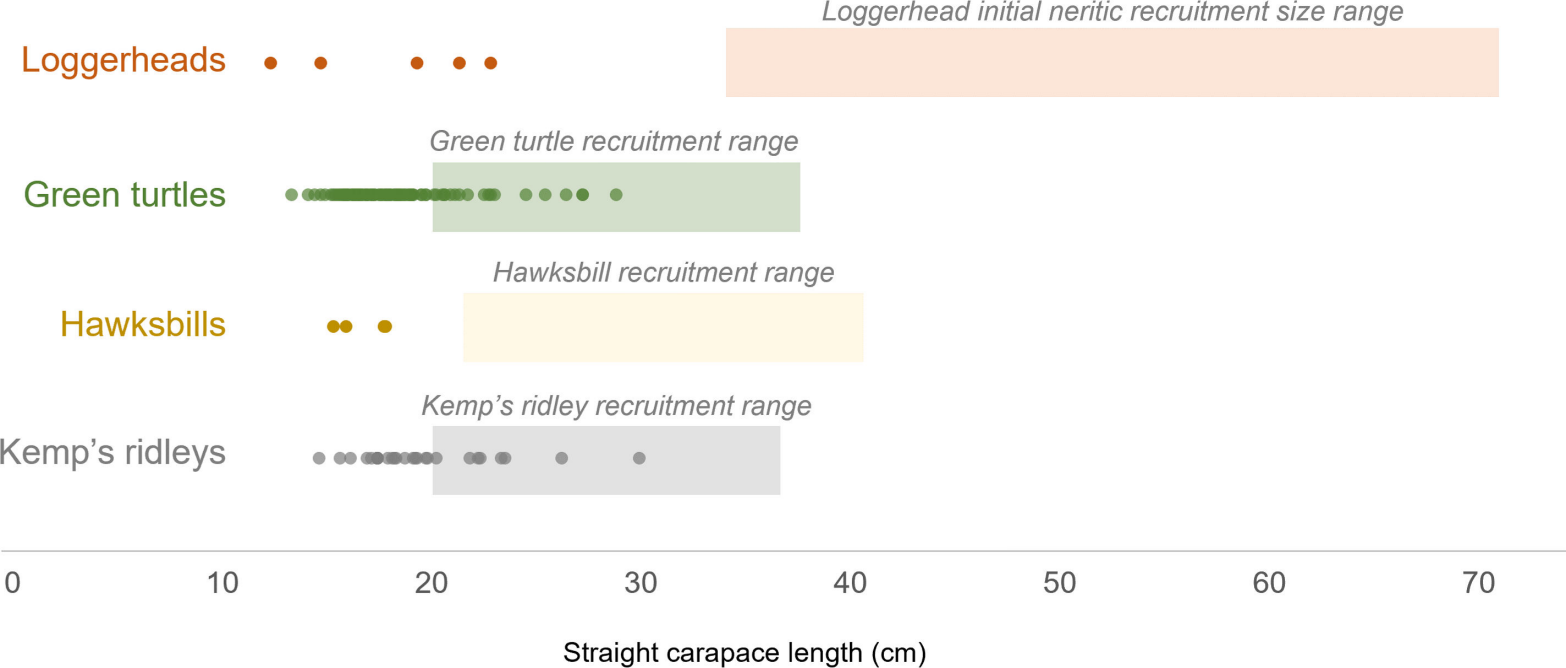
The sizes of dispersal-stage sea turtles sampled in the Gulf of Mexico (points) overlaid with each species’s potential size range at initial recruitment to neritic habitats along the southeastern United States (shaded boxes) based on previous observations in the region ([32,38,42,43,48,52,68–80]; see electronic supplementary material, table S1), not presented as a specific size cutoff. The largest green turtles and Kemp’s ridleys we sampled are within the size range that has been observed in shallow coastal habitats.

Between 2011 and 2022, we sampled 131 small juvenile sea turtles of four species: green turtles (*n* = 94), Kemp’s ridleys (*n* = 28), loggerheads (*n* = 5) and hawksbills (*n* = 4). All turtles were observed and captured at the sea surface. Straight carapace lengths (SCL) ranged from 12.3 to 29.9 cm with an average of 18.6 cm (s.d. 3.1; figure 1). Turtle weights ranged from 0.3 to 2.65 kg with an average of 0.9 kg (s.d. 0.5; electronic supplementary material, figure S1). The hawksbills encountered were generally the smallest, with an average SCL of 16.7 cm (s.d. 1.3) and weight of 0.7 kg (s.d. 0.1). For the remaining species, average green turtle SCL was 18.4 cm (s.d. 2.9) and average weight 0.8 kg (s.d. 0.4); average Kemp’s ridley SCL was 19.5 cm (s.d. 3.3) and weight 1.1 kg (s.d. 0.5); and average loggerhead SCL was 18.1 cm (s.d. 4.4) and weight 1.1 kg (s.d. 0.6). None of the loggerheads approached the estimated minimum size of nearshore recruitment around 40 cm SCL (figure 1; electronic supplementary material, table S1). Similarly, no hawksbills were within a 20−30 cm coastal recruitment size; however, 19 of the green turtles and of eight Kemp’s ridleys did fall within a size range of potential recruitment to nearshore habitats (figure 1; electronic supplementary material, table S1).

### (b) Tracking data

We released groups of turtles and drifters together over 37 deployments, with a total of 114 PTT-outfitted turtles and 64 paired drifters. Satellite-tracked turtles consisted of green turtles (*n* = 79), Kemp’s ridleys (*n* = 26), loggerheads (*n* = 5) and hawksbills (*n* = 4). The average turtle tracking duration was 37 days (s.d. 21.9). Green turtles had shorter tracking durations with an average of 31 days (s.d. 17), probably due in part to quicker tag loss from their smooth scutes [20]. Kemp’s ridleys had an average tracking duration of 45 days (s.d. 30), loggerheads averaged 61 days (s.d. 11) and hawksbills averaged 70 days (s.d. 37).

### (c) State-space modelling

**Figure 2.**
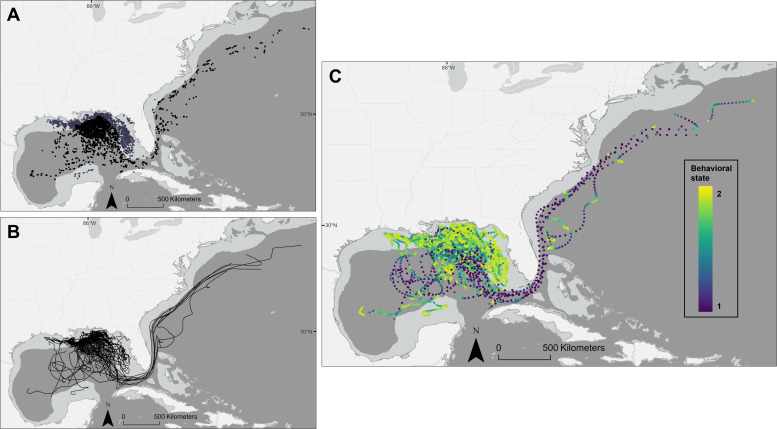
Juvenile sea turtle movements of 114 individuals were tracked from capture locations in the northeastern Gulf of Mexico. Land is shown in light grey, the continental shelf in medium grey, and depths >200 m in dark grey. (A) Argos locations for all tracked individuals. Black points indicate locations off the continental shelf, while purple points indicate locations over the shelf. (B) Interpolated pathways from the correlated random walk model for 112 individuals with tracking durations > 12 h. (C) State-space model estimates at each interpolated point. Dark purple points correspond with state estimates closer to the fast, directed state 1, while green and yellow points represent values approaching the slower, sinuous state 2.

**Figure 3.**
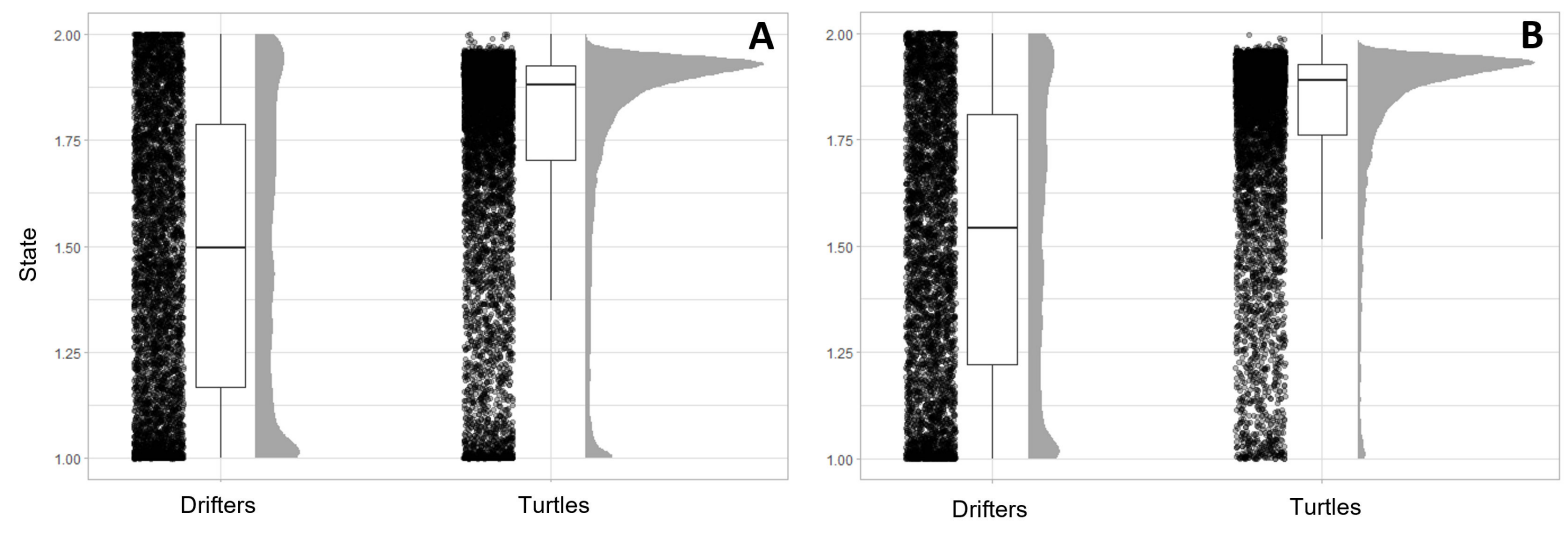
Distribution of estimated states for drifters and turtles based on (A) the full modelled dataset and (B) the dataset without drifters and turtles that exited the Gulf of Mexico via the Gulf Stream. Black points represent all data points, while the grey plots summarize the distribution of points between states 1 (quick, directed) and 2 (slow, sinuous). The proportion of points near 1 decreases when turtles travelling in the Gulf Stream are removed (B).

We interpolated positions for 112 turtles at 12 h intervals (figure 2). Turtles trended more towards state two movements than drifters, which had nearly even distribution of state estimates (figure 3). However, estimated turtle states did not approach values >1.95 as often as drifters.

**Figure 4.**
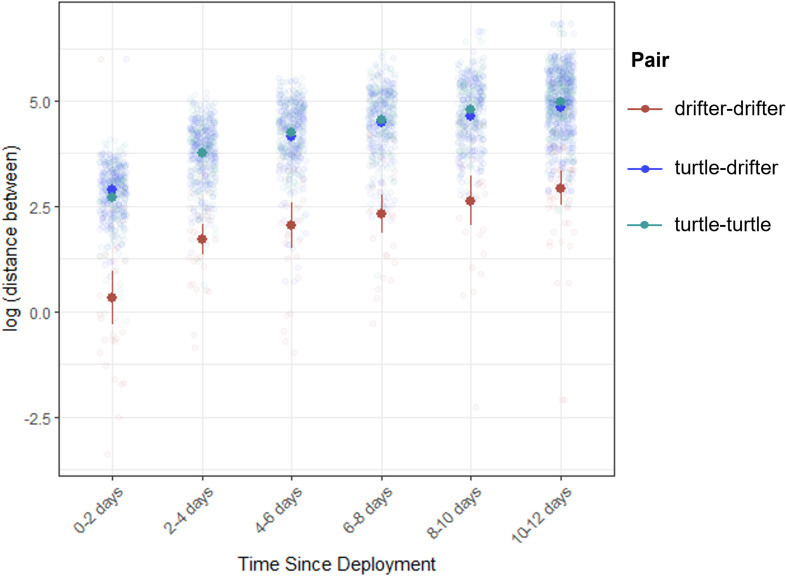
Log-transformed distances between drifters and turtles that were released together. Over the first two weeks post-deployment, drifters diverged less from other drifters than turtles diverged from either other turtles or drifters.

Drifters remained in closer proximity to other drifters than they did to turtles (figure 4). The average distance between drifters released in the same deployment was 15 km in 0−2 days, 9 km in 2−4 days, 16 km in 4−6 days and 39 km by 10−12 days. By contrast, the average distance between turtles and drifters released together was 22 km in 0−2 days, 53 km in 2−4 days, 85 km in 4−6 days and 177 km in 10−12 days. Similarly, the average distance between turtles and turtles was 19 km in 0−2 days, 54 km in 2−4 days, 85 km in 4−6 days and 185 km in 10−12 days.

### (d) Movement patterns

Of the 131 turtle encounters and captures, 60 occurred over the continental shelf (electronic supplementary material, figure S2). Once outfitted and released with PTTs, 89 individuals travelled over the shelf during the tracking period (figure 2). For SSM data at 12 h increments, 3168 of 8385 turtle locations occurred over the shelf (37.8%), while 2467 of 4970 drifter locations occurred over the shelf (49.6%; electronic supplementary material, figure S3). The turtle tracking data occurred between mid-May and December across years; therefore, we focused on environmental data between those dates for the GAM analysis comparing temperatures associated with turtle and drifter locations. Sea surface temperature did not differ between turtle and drifter locations, indicated by the similar splines for both groups in the analysis (electronic supplementary material, figure S4).

In terms of behavioural states, there was a higher frequency of positions with state estimates >1.9 among turtles over the shelf than off the shelf (electronic supplementary material, figure S5). The increased frequency of lower state estimates approaching the fast, directed state 1 off the shelf is probably due in part to entrainment in currents, while state estimates approaching the slow, sinuous state 2 on the shelf could indicate recruitment to shallow coastal habitats and restricted area search therein or alternatively a slowing and turning behaviour to avoid the coast.

**Figure 5.**
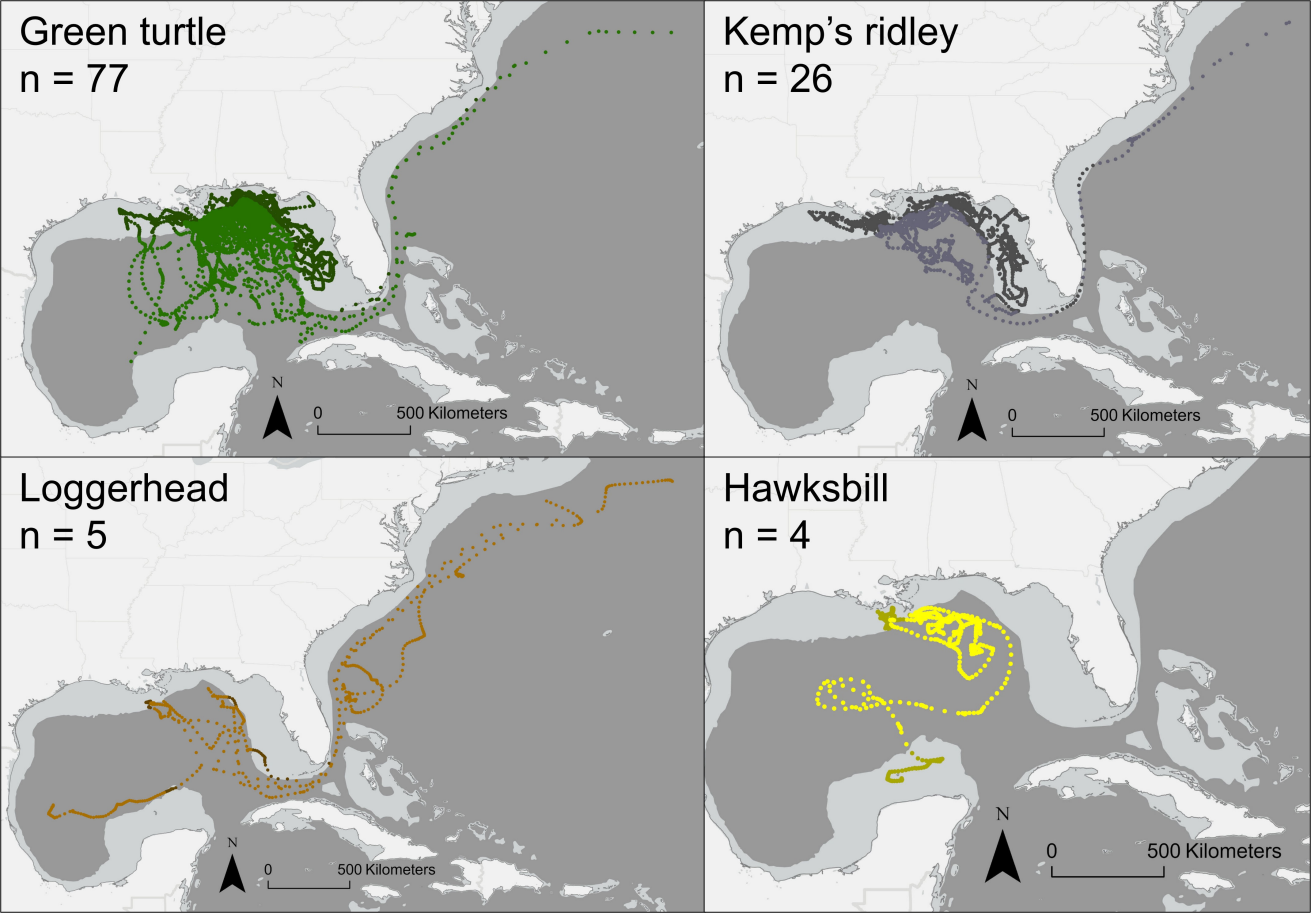
Interpolated points at 12 h intervals by species from the correlated random walk model dataset (*n* = 112). The light grey region shows the continental shelf. For each species, a colour shift to darker points illustrates positions over continental shelf.

There were species differences in continental shelf occupancy (figure 5). Kemp’s ridleys tended to remain over the continental shelf more than other species, with 64.9% of positions over the shelf, and just four of 26 tracked Kemp’s ridleys entering the centre of the Gulf of Mexico. In contrast, only 9.6% of loggerhead positions occurred over the shelf.

Nine tracked turtles followed the path of the Gulf Stream around the southern end of Florida and out of the Gulf of Mexico to the western North Atlantic (figure 5): 4 of 5 loggerheads, 4 of 79 green turtles, and 1 of 24 Kemp’s ridleys. None of the four tracked hawksbills exited the Gulf of Mexico during the tracking period. The low proportion of green turtles tracked outside the Gulf may relate to tag attachment longevity; green turtles tracked exiting the Gulf of Mexico had longer tracking durations averaging 53 days (range: 32−75 days) than the 31 day species average. Two loggerheads and one green turtle exited the Gulf Stream current eastward near Cape Canaveral. The green turtle ceased transmission soon after, while both loggerheads returned to the Gulf Stream within a month. The average time for turtles to reach the east coast of the United States was 34 days (s.d. 10; *n* = 9). The remaining 105 tracked turtles spent the duration of their tracking period within the Gulf of Mexico. The average time for drifters to follow the Gulf Stream around the tip of Florida to the east coast of the United States was longer at 77 days (s.d. 29; *n* = 4). No drifters deployed from capture sites off Destin, Florida, left the Gulf of Mexico, while six turtles from the same deployments exited the Gulf and travelled up the east coast of the United States as far as 40°N latitude. No turtles or drifters deployed from capture sites in the eastern Gulf of Mexico left the Gulf.

### (e) Potential ontogenetic habitat shifts

As turtles approached the coast, movements generally slowed and turning increased (nearer to behavioural state 2). We focused on a subset of turtle locations that occurred within 20 km of a coastline to assess possible nearshore recruitment, as this is a plausible distance that environmental cues could alert turtles that they were in or near neritic habitat [81,82]. Of the 114 tracked turtles, 32 travelled within 20 km of shore during their tracking period (28%): 17 of 79 green turtles, 12 of 26 Kemp’s ridleys, 2 of 4 hawksbills and 1 of 5 loggerheads. The single loggerhead approached a coastline as it rounded the southern tip of Florida presumably travelling with the Gulf Stream and subsequently continued with the current to enter the western North Atlantic, which was similar for one Kemp’s ridley and two green turtles. Three Kemp’s ridleys and one green turtle were still within 20 km of a coastline when transmissions ceased. Nine green turtles entered coastal waters near Louisiana, five along the Gulf coast of Florida (one remained near-shore when transmissions ended), and one near the northeast coast of Cuba. Nine Kemp’s ridleys also entered coastal waters near Louisiana, including two that remained through the end of tag transmission, and one that later entered coastal waters near Panama City, Florida. The highest modelled states (≥1.95) among coastal individuals were two Kemp’s ridleys turning potentially to avoid the coast near Grand Isle, Louisiana. Two additional Kemp’s ridleys entered coastal waters along the Gulf coast of Florida (including one that remained by the end of transmission). The two hawkbills that entered coastal waters were near the coasts of Louisiana and Scorpion Reef, Mexico.

Of the turtles tracked within 20 km of shore (*n* = 32), five were >25 cm SCL, a size at which coastal recruitment may be expected (figure 1, electronic supplementary material, table S1). Two of these were green turtles within 20 km of Louisiana for 3−7 days before both travelled south over 700 km through oceanic waters toward the Bay of Campeche. We tracked four turtles within 20 km of shore in areas where no drifters came within 20 km of the coast, which may indicate intentional recruitment to neritic habitats. However, one of these was a green turtle that approached the coast of Cuba, and subsequently followed the path of the Gulf Stream along the east coast of the United States where transmissions ended north of Cape Hatteras, North Carolina. The remaining three turtles in this category include a Kemp’s ridley (SCL 26.2 cm) and green turtle (SCL 25.4 cm) that approached the coast of Florida, and a hawksbill that travelled to Scorpion Reef, Mexico (SCL 17.8 cm). No turtles appeared to strand during the tracking period or were subsequently reported as stranded.

Conversely, 33 of the 64 drifters washed up along the United States coast in the states of Florida (*n* = 17), Louisiana (*n* = 9), Alabama (*n* = 6) and Mississippi (*n* = 1). Three of the six Alabama-beached drifters washed up on Dauphin Island. Of the Florida-beached drifters, 13 washed up along the Panhandle, one on Key West and three on the east coast south of Cape Canaveral. One drifter experienced a 58 day transmission gap before transmitting again near Tamaulipas, Mexico, an important area for Kemp’s ridley and green turtle nesting. Similarly, another drifter transmitted a final location from approximately 200 km east of Tamaulipas. In total, 47 of the 64 drifters came within 20 km of shore (73.4%).

## 4. Discussion

We present the largest dataset of wild-caught ‘lost years’ sea turtle behaviour to date, providing valuable insight into this understudied life stage. These data include the first satellite tracks for wild-caught hawksbills and loggerheads of this size. Our results establish the waters over the West Florida Shelf as an important habitat for dispersing juvenile sea turtles in the Gulf of Mexico. Our data also indicate that traditional definitions of ‘oceanic-stage’ juveniles [5,55] may be a misnomer, oversimplifying their ecology. A behaviourally descriptive term like ‘dispersal-stage’ is more accurate and better represents behaviour at this life stage. Additionally, dispersing turtles in the present study show evidence of directed swimming behaviour when compared with drifters, particularly when near the coast. Building on Putman & Mansfield [21], these data are valuable for assessing risk related to anthropogenic impacts including oil spills, fisheries interactions and climate change.

### (a) Dispersal-stage movement patterns

Previous juvenile sea turtle dispersal studies often focused on hatchlings entering the North Atlantic from Costa Rica, Bermuda or the southeastern United States [10,11,14,83]. As more turtles are tracked from other regions, such as the Gulf of Mexico [21] and South Atlantic [19], we find that assumptions once held for the sea turtle ‘lost years’, primarily based on loggerheads dispersing directly into the Gulf Stream, do not hold for all species or those dispersing from rookeries in different regions and ocean basins [16]. Mansfield *et al*. [19] tracked laboratory-reared loggerheads off the coast of Brazil; none entered the interior of the South Atlantic Subtropical Gyre like their northern counterparts [18], as many remained close to the coastline, and some travelled north into the Caribbean and across the equator into the North Atlantic Ocean [19]. The specifics of sea turtle dispersal behaviours may be closely related to both oceanographic and geographic constraints of the basins they occupy [84]. For example, in areas like the Gulf of Mexico and Mediterranean Sea, oceanic habitats and broad neritic habitats are in relatively close proximity and turtles may spend their entire lives within a single ocean basin. By contrast, turtles in some regions may move between ocean basins as they disperse (e.g. loggerheads from Brazil [19] and green turtles from rookeries in the western Indian Ocean [29,85]).

Sea turtles have capacity to navigate even at this early life stage [21,86]. Building on work by Putman and Mansfield *et al*. [21], the differing movement patterns between oceanographic drifters and turtles (figures 3 and 4) provide further evidence that turtles do not solely passively drift. While over the continental shelf, turtles did not approach shore as closely or strand on shore like drifters. One mechanism that may guide this behaviour is chemical cues; a study in Brazil found that loggerhead hatchlings choose to swim towards oceanic water preferentially over coastal water [81]. The increased frequency of higher behavioural states among turtles over the shelf (electronic supplementary material, figure S4), often labelled as ‘area restricted search’ or linked to recruitment, is more likely to reflect slowing swim speeds and turning away from shore. There was also a high frequency of behavioural state estimates closer to two off the continental shelf, which could reflect behaviour in and around *Sargassum*, or searching for suitable floating habitat in areas where *Sargassum* was not abundant or scattered due to high winds and seas. State-space model results should be interpreted carefully, as they are based solely on the speed and turning radius characteristics of tracking data and can easily be mis-defined. Future work comparing behavioural states to *Sargassum* presence/absence data will more clearly define these movements.

Contrary to previous dispersal-stage tracking studies in the Gulf Stream current [18,20], turtles in this study that departed the Gulf Stream towards the Sargasso Sea later returned to the Gulf Stream. The turtles we tracked reached the Gulf Stream at a different time of year and may have entered a different part of the current than the previous studies [18,20]. Mansfield *et al*. [20] documented nine green turtles leaving Gulf Stream south of Cape Hatteras, North Carolina, while six diverted north of Cape Hatteras. This region of the Gulf Stream is highly energetic, and eddies can detach from the main current and move either northward as warm-core rings or southward as cold-core rings [87]; given the variability in oceanographic conditions, differences in turtle trajectories between this study and Mansfield *et al*. [18,20] may be due to Gulf Stream dynamics rather than turtle behaviour [23]. On the other hand, there may also be differences between captive-reared turtle movements compared with wild-caught and released individuals.

Three of the four drifters carried by the Gulf Stream to the east coast of the United States washed up south of Cape Canaveral, Florida, while none of the nine turtles that travelled along the eastern US coast stranded on shore. If turtles orient to stay within currents that delineate the North Atlantic Subtropical Gyre, the three turtles that left the eastern edge of the Gulf Stream may have been attempting to avoid the US coast; even intermittent active orientation and directed swimming behaviour could factor into navigating away from shore [22,86,88]. Similarly, drifters that exited the Gulf of Mexico took longer than turtles to do so; turtles may be orienting to stay entrained in currents [86] or avoiding chemical cues from shore [81].

### (b) Ontogenetic shifts

Nearly 38% of the location points among the turtles tracked in this study occurred over the continental shelf. Many of these movements included later turns away from the coast and back out to deeper water. Our assessment of turtle movements within 20 km of shore that did not overlap with similar oceanographic drifter movements identified three individuals that may be beginning the process of recruiting to nearshore habitats, though tracking durations were too short to assign with certainty. A Kemp’s ridley (SCL 26.2 cm) and green turtle (SCL 25.4 cm) were within the range of sizes previously observed in neritic habitats, while the hawksbill that travelled to Scorpion Reef, Mexico (SCL 17.8 cm) was smaller than expected recruitment size (figure 1). The tracking devices in this study did not collect depth or dive data; it is not clear based on telemetry and SSM data alone whether nearshore behaviour shifted from surface-dwelling opportunistic foraging to more benthic foraging typically associated with ‘neritic’ juvenile turtles. While PTT transmissions may have ended due to tag loss related to interactions with benthic habitats, other causes include shedding tags as turtles grow, biofouling of solar panels, reduction in charging efficiency if turtles spend more time at depth or mortality [17,18,20]. Future work that includes dive data will better discriminate whether turtle movements close to shore include interactions with benthic environments.

Among our tracked loggerheads, none approached shore with the high behavioural states expected for neritic recruitment. This is not surprising, given that loggerheads begin recruiting to neritic habitats at larger sizes than the turtles we observed (figure 1). We reiterate that our loggerhead sample size is limited (*n* = 5), and additional tracking data may reveal different patterns. Loggerheads may take longer to recruit to neritic habitats due to natal homing prior to reaching maturity, perhaps more common in this species than others [89]. In a neritic developmental habitat on the east coast of Florida, the largest estimated loggerhead rookery contributions were from nearby rookeries in Florida [90]. On the other hand, mixed stock analyses of post-dispersal juvenile green turtles [91–95] and hawksbills [77,78,96] generally estimate contributions from rookeries located farther from developmental foraging sites. This may be one factor contributing to the plasticity observed in loggerhead recruitment to neritic habitats [43,44], shifting back and forth between oceanic habitats after a longer initial period away from shore than other species. Therefore, the smaller, and presumably quicker recruiting species may have post-dispersal mixed stock composition driven more by current transport and frontal boundaries than loggerheads. As a result, the approach of incorporating distance (e.g. 92,95) or transport probabilities into mixed stock analyses [29] may be more informative for the other offshore-to-nearshore dispersing species than for loggerheads.

Of course, we are finding that there are exceptions to every rule and assumption. The distribution of genetic variation at hawksbill foraging sites mirrors particle simulations, but not entirely, with swimming behaviour a likely factor [28]. Natal homing before neritic recruitment has also been suggested for hawksbills [97], which may explain the directed movements of the hawksbill in the current study toward Scorpion Reef, Mexico. As with the loggerhead data, our hawksbill tracking data are limited (*n* = 4), yet the first of its kind for this life stage. Mansfield *et al*. [19] documented dispersal-stage loggerhead turtles travelling north from Brazil and crossing the equator before entering the Caribbean Sea, and loggerheads with haplotypes linked to rookeries in Brazil have been observed in neritic habitats in North Carolina, Florida and the Azores [98–100]. These sites may be convenient stopovers during the long loggerhead dispersal period before shifting to developmental sites closer to their natal beach, or longer duration coastal recruitment away from natal areas.

Natural selection favours hatchlings from rookeries where currents are favourable for survival [2] and many major rookeries occur near strong currents such as the Kuroshio Current off Japan, East Australian Current off Australia, Ras al Hadd Jet off Oman, Agulhas Current off South Africa, Brazil Current off Brazil, Yucatan Current off Mexico, and Gulf Stream off the United States [101]. In a related way, Kemp’s ridley nest site densities correlate with areas that support connectivity with foraging sites [102]. Two drifters released in this study eventually made their way towards Tamaulipas, Mexico, close to high-density Kemp’s ridley nesting areas. The oceanography of the region may not only favour dispersal away from shore immediately after hatching but also to later return to natal sites.

### (c) Conservation implications

The turtles in this study were captured in United States waters but may originate from rookeries outside the United States [29], an important consideration in the context of species conservation and management units [103]. Additionally, these turtles are transient—moving either with or independent of dominant currents, as well as exhibiting directed movement and active swimming [21]. Sea turtles in this life stage are too small to be reliably detected by aerial surveys, limiting the ability to monitor distributions and abundance. Results from [16,29] combined with tracking data from the present study show that the northern Gulf of Mexico and West Florida Shelf provide important developmental habitat for turtles originating from rookeries throughout the wider Caribbean and Gulf of Mexico. Although our sampling locations were limited to the northeastern Gulf of Mexico, which introduces an element of sampling bias, the West Florida Shelf is emerging as an important ontogenetic transition zone, particularly for Kemp’s ridley and green turtles. This is further supported by well-documented ‘neritic’ post-dispersal developmental habitats for these species along Florida coast in the eastern Gulf of Mexico [31–38].

Perhaps one of the most important conservation implications of this study, and the cumulative tracking work focusing on the sea turtle ‘lost years’, is the need to shift our terminology and management perspective from place-based life-stage definitions (e.g. oceanic, surface-pelagic, neritic and coastal) to behavioural definitions. We suggest that a more appropriate term for the sea turtle ‘lost years’ life stage is *dispersal stage*. As documented in the present study and by Mansfield *et al*. [19] in the South Atlantic, small juvenile sea turtles do not exclusively occur in ‘oceanic’ waters > 200 m depth off the continental shelf.

### (d) Future needs and outstanding questions

As we deploy more satellite tags on dispersal-stage turtles, we are learning that not all turtles adhere to early assumptions about behaviour and dispersal patterns [18–21]. More data are needed from wild-caught-and-released turtles in regions outside of the western North Atlantic (e.g. [104,105]) to understand behaviour in different ocean bodies with different circulation patterns. In the North Atlantic, dispersal-stage turtles associate with floating *Sargassum*. However, in regions where *Sargassum* is not present or prevalent, how do dispersal-stage turtles reliably locate food, refuge, warmth and transport during a critical period of growth? The presence or absence of this resource probably influences survival rates and energy expenditures and possibly regional population trajectories.

Within regions where *Sargassum* is prevalent, more data are needed on how and when turtles associate with the algal habitat. *Sargassum* is transient; large mats or windrows can break up with a shift in winds or sea state. While Mansfield *et al*. [18,20] demonstrated that dispersal-stage turtles probably spend most of their time at the sea surface, finer scale data are needed to investigate how individuals locate *Sargassum* habitats and the shifts in dive behaviour as turtles grow and begin transitioning to nearshore habitats.

Cheloniid ontogenetic shifts from a dispersal stage to benthic foraging appear to be more of a transition than a sudden and permanent shift, at least among turtles in the North Atlantic. There are also differences in timing, particularly for loggerheads versus other species, which may correspond with differences in natal homing prior to approaching neritic developmental habitats. We are currently in a period of rapid environmental change, and the present study serves as an important baseline of juvenile sea turtle distributions and behaviour for building conservation plans [106]. Whether Atlantic sea turtle species will adapt to quickly changing conditions or current patterns [107] remains to be seen. Increased frequency or severity of hurricanes with climate change could reduce *Sargassum* habitat by scattering and sinking the floating algae [108,109], although these sea turtle species and others also occur in ocean basins without surface *Sargassum*. There is also evidence of declining growth rates among juvenile turtles in the region [110], which may correspond with smaller turtles recruiting from the dispersal stage to benthic habitats closer to shore. By uncovering the secrets of the sea turtle ‘lost years’, we are better equipped to establish effective conservation and management plans to ensure their persistence in the future.

## Data Availability

The raw telemetry data from oceanographic drifters and PTTs affixed to individual turtles, along with R script for state space models, are available on the Dryad Digital Repository [111]. Additional electronic supplementary material containing tables S1–S3 and figures S1–S5 is available online [112].
